# Editorial: Predicting, managing, and minimizing mycotoxicosis in farm animals

**DOI:** 10.3389/ffunb.2024.1519411

**Published:** 2024-11-15

**Authors:** Jesús Adonai Maguey-González, Juan D. Latorre, Sergio Gomez-Rosales, Abraham Mendez-Albores

**Affiliations:** ^1^ Department of Poultry Science, University of Arkansas, Fayetteville, AR, United States; ^2^ Centro Nacional de Investigacion Disciplinaria en Fisiologia y MejoramIento Animal (CENID)-Instituto Nacional de Investigaciones Forestales, Agricolas y Pecuarias (INIFAP), Querétaro, Mexico; ^3^ Unidad de Investigación Multidisciplinaria L14 (Alimentos, Micotoxinas, y Micotoxicosis), Facultad de Estudios Superiores (FES) Cuautitlán, Universidad Nacional Autonoma de Mexico (UNAM), Cuautitlán Izcalli, Estado de México, Mexico

**Keywords:** mycotoxins, mycotoxicosis, adsorbents, animal diseases, animal health

## 1 Introduction

Mycotoxins are fungal secondary metabolites with toxic properties that significantly threaten human and animal health. Over 500 distinct mycotoxins have been identified and chemically characterized, primarily from three genera: *Aspergillus*, *Penicillium*, and *Fusarium*. When ingested, mycotoxins can lead to a range of health issues depending on the specific mycotoxin, exposure level and time. Thus, mycotoxicosis is a toxic condition that occurs when animals or humans ingest or inhale fungal toxins. In the poultry industry, this condition can adversely affect animal health and productivity, resulting in reduced growth rate, impaired feed efficiency, immune suppression, reproductive disorders, organ damage, and in extreme cases, death.

The need to predict, manage, and minimize mycotoxicosis is crucial in various sectors, including agriculture, food safety, and veterinary medicine. By harnessing these recent advances and focusing on comprehensive preventive measures such as accurate detection, predictive modeling, detoxification, and nutritional strategies, we can effectively combat mycotoxicosis, safeguard animal health, and ensure the production of safe and nutritious food for human consumption. Thus, promising approaches to reduce mycotoxin levels in animal feed and improve overall safety include using adsorbent materials (organic, inorganic, and hybrid), enzymatic treatments, and biological agents among others.

In this context, Gómez-Osorio et al. reviewed the relationship between mycotoxins and coccidiosis in poultry, emphasizing their co-occurrence and interaction as well as their effects on poultry health and productivity. This review underlines the need for effective management strategies to mitigate the combined risks of mycotoxins and coccidiosis and advocates for a holistic approach that should also include the following aspects: rigorous feed management, disease prevention measures, and regular monitoring to maintain the health and productivity of poultry against these significant challenges.

Moreover, Kappari et al. summarized current research on the role of microRNAs (miRNAs) in certain farm animal diseases, including mycotoxicosis. Through a critical literature review, the authors focused on the impact of miRNAs in the pathogenesis of viral and bacterial infections and mycotoxicosis. Overall, the review highlighted the potential of miRNAs as biomarkers for early disease detection and intervention, which could significantly benefit farm animal health and improve productivity.

Furthermore, Maguey-Gonzalez et al. evaluated the efficacy of humic acids derived from worm compost in counteracting the toxic effects of aflatoxin B_1_ (AFB_1_) in young turkey poults. This study presents a practical approach to the utilization of non-nutritive adsorbent materials, which can effectively bind AFB_1_ and inhibit its absorption in the gastrointestinal tract, thereby minimizing the toxic impact on poultry and decreasing the risk of this fungal metabolite entering poultry products such as meat and eggs. The findings of this research suggest that humic acids could serve as a valuable natural additive to tackle mycotoxin-related challenges in poultry production by improving overall productivity and health.

